# *BRCA1* Promoter CpG Methylation in Breast Cancer: A Pilot Study in African Women

**DOI:** 10.3390/genes17040407

**Published:** 2026-03-31

**Authors:** Tarryn Willmer, Mpoi Makhetha, Ayesha Rasheed Shaik, Lawrence Mabasa, Ines Buccimazza, Colleen Aldous

**Affiliations:** 1 Biomedical Research and Innovation Platform, South African Medical Research Council, Tygerberg 7505, South Africa; ayesha.shaik@mrc.ac.za (A.R.S.); lawrence.mabasa@mrc.ac.za (L.M.); 2 Centre for Cardio-Metabolic Research in Africa, Division of Medical Physiology, Faculty of Medicine and Health Sciences, Stellenbosch University, Tygerberg 7505, South Africa; 3 Division of Cell Biology, Department of Human Biology, Faculty of Health Sciences, University of Cape Town, Cape Town 7700, South Africa; 4 Department of Clinical Medicine, Nelson R. Mandela School of Medicine, University of KwaZulu-Natal, Durban 4001, South Africa; mpoi.florence@gmail.com (M.M.); aldousc@ukzn.ac.za (C.A.); 5 Department of Oncology, Inkosi Albert Luthuli Central Hospital, Durban 4091, South Africa; ines@orthoserve.co.za

**Keywords:** epigenetic biomarkers, breast cancer, *BRCA1*, DNA methylation, Africans

## Abstract

**Background**: Breast cancer susceptibility gene 1 (*BRCA1*) is a pivotal regulator of DNA repair, and its loss through germline mutations is strongly linked to the development of aggressive breast cancers with characteristic clinical and pathological features. Beyond genetic disruption, epigenetic silencing via promoter hypermethylation has emerged as a non-mutational mechanism of tumour suppressor inactivation and a potential biomarker for guiding therapeutic decisions. Here, we investigate *BRCA1* promoter methylation, its impact on gene expression, and its association with clinicopathological features in a cohort of African women with breast cancer. **Methods**: Matched tumour and adjacent normal tissues from 27 Black African women with breast cancer were analysed for *BRCA1* promoter methylation and gene expression using bisulfite pyrosequencing and quantitative real-time PCR. Associations with clinicopathological variables were assessed using Spearman’s correlation analyses. **Results**: Five CpG sites within the *BRCA1* promoter were significantly hypermethylated in breast tumours compared with matched adjacent normal tissues and showed an inverse association with *BRCA1* mRNA expression. Elevated promoter methylation was enriched in hormone receptor-negative and triple-negative breast cancer subtypes and was not influenced by neoadjuvant chemotherapy. *BRCA1* promoter methylation occurred independently of *BRCA1* mutational status. No significant associations were observed between *BRCA1* methylation and age, body mass index, smoking status, or alcohol consumption. **Conclusions**: Our findings provide evidence of *BRCA1* epigenetic silencing in breast tumours from African women, particularly within aggressive hormone receptor-negative subtypes. These results suggest that *BRCA1* promoter methylation may represent a clinically informative biomarker for patient stratification and highlight the importance of validation in larger, population-representative cohorts before clinical translation.

## 1. Introduction

Breast cancer is the most commonly diagnosed malignancy among women worldwide and in Africa, and it remains a leading cause of cancer-related mortality. In 2022, approximately 2.3 million new cases were reported worldwide, with over 670,000 deaths attributed to the disease [1]. The global burden of breast cancer is expected to rise substantially, with annual new diagnoses projected to exceed 3 million by 2040, disproportionately affecting low- and middle-income countries [2]. These trends underscore the growing public health urgency of breast cancer, particularly in regions such as sub-Saharan Africa, where healthcare resources and molecular diagnostic capacity remain limited.

Despite major advances in molecular characterisation and targeted therapy, population-specific data to guide early detection and personalised treatment remain scarce, particularly for women of African ancestry. In Africa, breast cancer represents a major public health burden, and although incidence is lower among Black African women than in Caucasians, women of African ancestry are more likely to present at a younger age, exhibit high-grade and aggressive phenotypes, and have poorer clinical outcomes [3,4,5,6]. These disparities may be explained by differences in tumour biology; however, there is a paucity of information on the factors driving the biology and clinical behaviour of breast cancer in women of African ancestry, who remain underrepresented in the literature.

Approximately 5–10% of breast cancers are hereditary and are commonly characterised by early-onset disease, high-grade histology, and a triple-negative phenotype [7]. Hereditary breast cancers are most frequently driven by pathogenic variants in tumour suppressor genes, particularly *Breast cancer susceptibility genes 1* and *2* (*BRCA1* and *BRCA2*), which confer lifetime breast cancer risks of up to 87% and 84%, respectively [7,8]. These variants typically result in loss of protein function through truncation or nonsense-mediated mRNA decay [9]. Consequently, *BRCA1/2* mutation status forms a cornerstone of clinical management, as tumours harbouring these alterations are highly sensitive to poly(ADP-ribose) polymerase (PARP) inhibitors such as olaparib and talazoparib [10]. These tools were initially designed for use in Europeans, and while rare mutations in *BRCA1/2* have been identified in African populations, these are not currently considered in routine testing [11]. As a result, there is concern that pathogenic variants present in African populations may be misclassified as benign or uninformative, potentially leading to inappropriate treatment decisions [12]. To combat this, studies are needed to identify novel breast cancer markers in ethnically diverse populations.

The majority of breast cancers do not arise solely from germline mutations but instead reflect complex interactions between genetic susceptibility, environmental exposures, and lifestyle factors [13,14]. The influence of these factors on tumour biology is largely mediated through epigenetic mechanisms, which regulate gene expression without altering the underlying genetic sequence [15]. DNA methylation is the best understood epigenetic mechanism and is defined as the covalent addition of a methyl group to cytosine residues within CpG dinucleotides [16,17]. Aberrant DNA methylation represents an early and central event in breast tumourigenesis, where promoter methylation can lead to transcriptional repression of tumour suppressor genes and has been widely implicated in cancer initiation, progression, and therapeutic response. As a result, dysregulated DNA methylation of several genes have been proposed as potential markers for the diagnosis of breast cancer, and may be useful in predicting response to treatment, recurrence, and overall survival [13,14,18]. Among these, *BRCA1* promoter hypermethylation has emerged as a potential mechanism of functional gene inactivation in sporadic breast cancer. *BRCA1* promoter hypermethylation has been reported in breast, ovarian, and pancreatic cancers and has been linked to aggressive tumour features and reduced responsiveness to endocrine therapies. [18,19,20,21,22]. This raises the possibility that patients with epigenetically inactivated *BRCA1* may benefit from therapies used for *BRCA1*-mutant tumours, such as PARP inhibitors; however, studies evaluating its predictive value for PARP inhibitor response have reported conflicting findings [18,22,23,24,25,26,27,28,29,30,31,32,33,34]. Moreover, the prevalence and clinicopathological significance of *BRCA1* promoter methylation in women of African ancestry remain largely uncharacterised, highlighting the need for further investigation in these populations.

The present study aimed to investigate the DNA methylation status of the *BRCA1* promoter in tumour versus normal adjacent tissue of a cohort of 27 Black African women recruited from the Inkosi Albert Lethuli Central Hospital in Kwa-Zulu Natal, South Africa, and further examined its association with gene expression and clinicopathological characteristics. We show that the *BRCA1* promoter was significantly hypermethylated in five interrogated CpG sites in tumours versus matched, normal adjacent tissue, which inversely correlated with mRNA expression levels and was independent of *BRCA1* mutation status. Importantly, elevated *BRCA1* promoter methylation was predominantly observed in hormone receptor-negative and triple-negative breast cancer (TNBC) subtypes and remained unaffected by neoadjuvant chemotherapy. We therefore propose that *BRCA1* promoter methylation could serve as a basis for developing epigenetic risk assessment tools and targeted intervention strategies, which are currently limited in this population.

## 2. Materials and Methods

### 2.1. Patient Selection

We recruited 27 Black South African patients diagnosed with operable primary ductal carcinoma in situ (DCIS) or invasive breast cancer who presented at Inkosi Albert Luthuli Central Hospital (IALCH), KwaZulu-Natal, South Africa. The study was approved by the Biomedical Ethics Research Committee (BREC/2019/00000613) and the South African Medical Research Council Human Research Ethics Committee (EC037-8/2021), and all women provided written informed consent. Women with stage IV disease or whose treatment did not include surgery were excluded. Patient data was obtained from the clinical files on enrolment, where demographic data comprised race, which was self-reported, body mass index (BMI), maternal age, parity, and age at menopause. Clinical data comprised age at diagnosis (dx), disease type, stage, tumour grade, *BRCA1/2* status, preoperative treatment, clinical response, family history, and comorbidities.

### 2.2. Tissue Collection and Pathological Analysis

The 27 pairs of fresh breast tumours and adjacent normal tissues were obtained from patients who underwent breast surgery at IALCH, KwaZulu-Natal, South Africa, between June 2021 and February 2022. Adjacent normal tissues were excised ~5 cm from the tumours and selected by a pathologist based on histopathological observations. The specimens were snap-frozen in liquid nitrogen and immediately stored at −80 °C until DNA and RNA extractions.

### 2.3. DNA Extraction

Genomic DNA was extracted from ~100 mg of tumours and adjacent normal tissues using the E.Z.N.A. Tissue DNA kit (Omega Bio-tek, Inc., Norcross, GA, USA) as recommended by the manufacturer. DNA purity and concentration were assessed using the Nanodrop Spectrophotometer (Nanodrop™ One, Thermo Fisher Scientific, Waltham, MA, USA) and Qubit fluorometer (Life Technologies, Carlsbad, CA, USA), respectively. The average of two measurements for each sample was used, where an A260/A280 ratio of ~1.8 and an A230/A260 ratio of ~2.0 were accepted as pure.

### 2.4. Pyrosequencing

#### 2.4.1. Primer Design

Forward, reverse and sequencing pyrosequencing primers, spanning CpG sites in the distal *BRCA1* promoter, were designed using the PyroMark Assay Design 2.0. The *BRCA1* sequence (GRCh38; Refseq ID: NM_007294.4) was downloaded from Ensembl [https://www.ensembl.org/index.html (accessed on 24 January 2023)] and six CpG sites spanning regions between -861 and -901 base pairs upstream of the *BRCA1* transcriptional start site on chromosome 17 were selected. The selected regions were chosen as they overlapped or flanked regions previously associated with breast cancer [35,36,37].

#### 2.4.2. Bisulfite Conversion

Bisulfite conversion of 1 µg of isolated DNA was conducted using the EpiTect^®^ Fast Bisulfite Conversion Kit (Qiagen, Hilden, Germany) according to the manufacturer’s recommendations. The PCR conditions were described previously, which were: 95 °C for 5 min, 60 °C for 10 min, 95 °C for 5 min, 60 °C for 10 min, and 4 °C for less than 20 h [38]. Bisulfite-treated DNA was desulphonated, washed, and eluted via a column-based method prior to use in subsequent PCR and pyrosequencing experiments.

#### 2.4.3. Pyrosequencing

*BRCA1* promoter methylation was assessed using pyrosequencing. PCR amplification of bisulfite-converted DNA was performed in a Veriti 96-well Thermal Cycler (Thermo Fisher Scientific) using the PyroMark^®^ PCR Kit (Qiagen, Valencia, CA, USA) according to the manufacturer’s instructions. The cycling protocol consisted of an initial denaturation at 95 °C for 15 min, followed by 45 cycles of 94 °C for 30 s, 56 °C for 30 s, and 72 °C for 30 s, with a final extension at 72 °C for 10 min. Amplification success and product size were verified by agarose gel electrophoresis.

Pyrosequencing was performed as previously described using a PyroMark^®^ Q96 ID pyrosequencer with PyroMark Q96 Gold reagents (Qiagen, Valencia, CA, USA) following the manufacturer’s instructions [38]. Bisulfite-converted PCR amplicons were denatured to single-stranded DNA, and biotinylated strands were captured using Streptavidin-coated beads. Sequencing primers were annealed to the single-stranded DNA by incubation at 80 °C for 2 min. Pyrograms were analysed using the PyroMark Q96 software, which calculated the percentage of methylation at each CpG site based on peak heights. To ensure assay reliability, methylated and unmethylated control DNA (EpiTect^®^ Test Control DNA, Qiagen) was included in every run. In addition, a no-template PCR control and a bisulfite conversion control were incorporated to monitor specificity and conversion efficiency. Any assay failing these quality control checks was repeated.

### 2.5. RNA Extractions

Total RNA was isolated from approximately 100 mg of breast cancer tissue and matched normal tissue using TRIzol™ Reagent (Invitrogen, Life Technologies, Carlsbad, CA, USA), followed by purification with the RNeasy^®^ Mini Kit (Qiagen, Valencia, CA, USA) according to the manufacturer’s protocols. RNA concentration was quantified with the Qubit RNA High Sensitivity (HS) assay kit on a Qubit 4 Fluorometer (Invitrogen, Thermo Fisher Scientific). RNA integrity was assessed using the Agilent 4200 TapeStation™ System (Agilent Technologies, Santa Clara, CA, USA), and RNA Integrity Numbers (RIN) were recorded for all samples.

### 2.6. Quantitative Real-Time PCR

Complementary DNA (cDNA) was generated from 1 μg of total RNA using the High-Capacity cDNA Reverse Transcription Kit (Applied Biosystems, Carlsbad, CA, USA) following the manufacturer’s instructions. Reverse transcription reactions were performed in duplicate, including both positive (+RT) and negative (–RT) controls. Reactions were carried out in a Veriti 96-well Thermal Cycler (Thermo Fisher Scientific, Waltham, MA, USA) under the following conditions: 25 °C for 10 min, 37 °C for 120 min, and 85 °C for 5 s. Synthesised cDNA was stored at −20 °C until use for qRT-PCR.

*BRCA1* mRNA expression was quantified using TaqMan Gene Expression Assays (Hs01556193_m1) with TaqMan Universal PCR Master Mix (Applied Biosystems, Carlsbad, CA, USA) according to the manufacturer’s protocol. Reactions were performed on a QuantStudio 7 Flex Real-Time PCR System (Thermo Fisher Scientific, Waltham, MA, USA). Relative gene expression levels were determined using the standard curve method and normalised to two validated endogenous reference genes. The stability of five candidate housekeeping genes, B2M (Hs00187842_m1), GUSB (Hs00939627_m1), PUM1 (Hs00472881_m1), RPLP0 (Hs00420895_gH), and SYMPK (Hs00191361_m1), was assessed in our sample set using geNorm, which identified RPLP0 and SYMPK as the most stable reference genes. These genes were subsequently used to normalise *BRCA1* expression in the qRT-PCR analysis. Samples with insufficient RNA quality or failed amplification were excluded from gene expression analysis.

### 2.7. Statistical Analysis

This study represents a hypothesis-driven exploratory analysis of *BRCA1* promoter methylation in breast cancer among Black South African women. The sample size of 27 participants was determined by the availability of matched tumour and normal tissue samples collected during the COVID-19 pandemic and aligns with similar pilot methylation studies designed to generate preliminary data [39]. Although relatively small, the cohort was sufficient to identify *BRCA1* methylation patterns and potential associations that can guide the design of larger, adequately powered studies. Given the limited sample size, adjustments for multiple testing were not applied; however, none of the participants reported smoking, and only two reported occasional alcohol consumption. Additionally, no significant correlations were observed between *BRCA1* methylation and age or BMI.

All statistical analyses were performed using GraphPad Prism version 10.6.1 (GraphPad Software Inc., San Diego, CA, USA). Data distributions were assessed using the Shapiro–Wilk test and are presented as mean and standard deviation (SD) for normally distributed data or median and interquartile range (25th–75th percentile) for non-normally distributed data. Comparisons of DNA methylation and gene expression between tumour and matched normal tissues were performed using paired Student’s *t*-tests for normally distributed data and Wilcoxon signed-rank tests for non-normally distributed data. Correlations between DNA methylation and clinicopathological parameters were evaluated using Pearson’s correlation for parametric data and Spearman’s rank correlation for non-parametric data. A *p*-value of <0.05 was considered statistically significant.

## 3. Results

### 3.1. Participant Characteristics

The demographic and clinical characteristics of the participants, along with histomorphological features of their tumours, are summarized in Table 1 and Table 2. The mean age at diagnosis was 49.6 ± 12.3 years, and the mean body mass index (BMI) was 32.5 ± 8.2 kg/m^2^, with the majority of women classified as overweight or obese. Most participants experienced menarche at 12 years or older and were multiparous, with an average maternal age of 22.1 years and a mean of 2.63 live births; six women reported previous miscarriages. Fifty-six percent of the cohort were premenopausal. The most common comorbidities were HIV infection (52%) and hypertension (29.6%), and 66% of women reported prior use of hormonal contraception.

Clinically, invasive ductal carcinoma was the predominant histological type (81.5%), and more than half of the participants (51.8%) presented with stage 3 disease. The most frequent molecular subtype was luminal B (59.2%), and 51.9% of tumours were grade II. A pathogenic variant in *BRCA1* was identified in one patient, whilst two patients harboured *BRCA2* mutations. Seventeen patients received neoadjuvant chemotherapy (NACT), of whom four achieved a near-total response, five had a partial response, and seven exhibited stable disease.

### 3.2. BRCA1 Methylation Analysis

**Figure 1:**
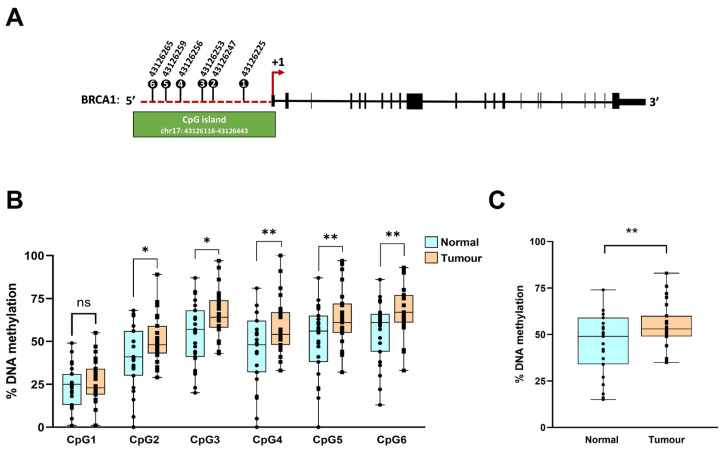
(**A**) Schematic diagram of the *BRCA1* gene, illustrating the six CpG sites analysed in the promoter region. The genomic coordinates of each CpG site are indicated. (**B**) Percentage methylation of individual CpG sites and (**C**) cumulative methylation levels of *BRCA1* in breast tumour versus normal tissue (n = 27 per group). Data represented as mean ± SD. * *p* < 0.05, ** *p* < 0.01, ns, not significant.

We analysed the DNA methylation status of six CpG sites within the *BRCA1* promoter using bisulfite pyrosequencing (Figure 1A). These CpG sites (CpG1–6), located at −861, −883, −889, −892, −895, and −901 base pairs upstream of the transcriptional start site, overlap or flank promoter regions previously implicated in breast cancer-associated epigenetic regulation [35,36]. Quantitative analysis demonstrated significantly higher methylation levels at CpG2, CpG3, CpG4, CpG5 and CpG6 in tumour tissues compared with matched normal breast tissue (Figure 1B). In addition, tumours exhibited significantly increased cumulative *BRCA1* promoter methylation across all six CpG sites (Figure 1C).

### 3.3. BRCA1 Methylation Associations with Clinicopathological Variables

**Figure 2:**
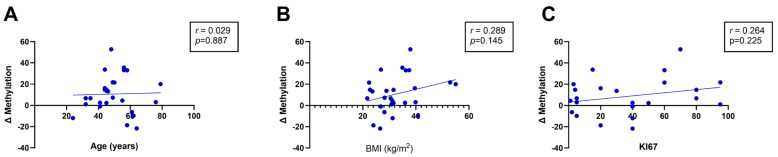
Spearman’s correlation analysis between the change (Δ) in *BRCA1* CpG methylation and (**A**) age, (**B**) body mass index (BMI), and (**C**) Ki67 proliferative index in tumour relative to matched adjacent normal tissues from South African women (n = 27 per group). Linear regression lines are shown for descriptive purposes only.

The tumour-specific methylation changes observed prompted further investigation into the association between *BRCA1* promoter methylation and clinicopathological variables. As shown in Figure 2, no significant correlations were observed between tumour-specific *BRCA1* methylation changes (Δ DNA methylation) and patient age (r = 0.029; *p* = 0.887), body mass index (BMI) (r = 0.289; *p* = 0.145), or tumour proliferative index (Ki67) (r = 0.264; *p* = 0.225). These findings suggest that, in this cohort, DNA methylation changes are not driven by age-related epigenetic drift, obesity, or tumour proliferative activity.

**Figure 3:**
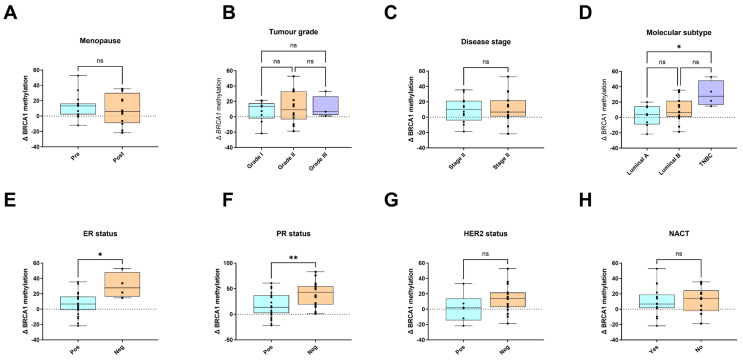
Association between the change (Δ) in *BRCA1* promoter methylation and clinicopathological characteristics in breast tumours from South African women (n = 27 per group). Δ *BRCA1* methylation was compared according to (**A**) menopausal status, (**B**) tumour grade, (**C**) disease stage, (**D**) molecular subtype, (**E**) oestrogen receptor (ER) status, (**F**) progesterone receptor (PR) status, (**G**) HER2 status, and (**H**) receipt of neoadjuvant chemotherapy (NACT). Data are presented as mean ± SD. Statistical significance was assessed using non-parametric tests as appropriate; * *p* < 0.05, ** *p* < 0.01, ns, not significant.

Similarly, no significant differences in Δ DNA methylation were observed with respect to age at menopause, tumour grade, or disease stage (Figure 3A–C). In contrast, significant differences were detected across molecular subtypes and hormone receptor statuses. Indeed, Δ DNA methylation levels were significantly higher in TNBC compared with luminal A tumours (Figure 3D). Likewise, ER-negative and PR-negative tumours exhibited significantly increased Δ DNA methylation relative to ER-positive and PR-positive tumours, respectively (Figure 3E,F). No significant associations were observed between Δ DNA methylation and HER2 status, nor between patients treated with neoadjuvant chemotherapy and treatment-naïve patients.

### 3.4. BRCA1 Expression Analysis

**Figure 4:**
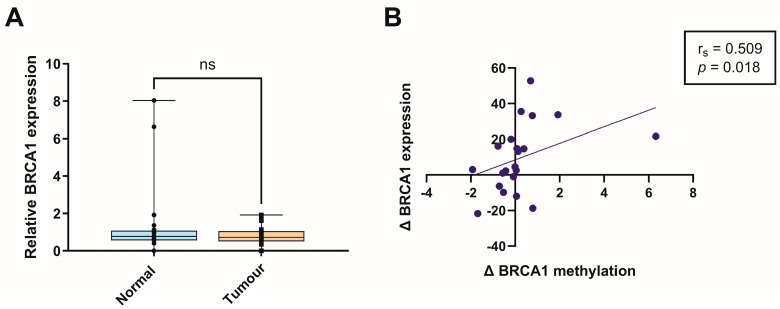
*BRCA1* expression analysis in tumour and matched normal breast tissues from South African women. (**A**) Relative *BRCA1* mRNA expression levels in tumours relative to matched adjacent normal tissues from South African women (n = 27 per group). Data are presented as mean ± SD; ns, not significant. (**B**) Spearman’s correlation analysis between the change (Δ) in *BRCA1* promoter methylation and the change (Δ) in *BRCA1* mRNA expression between tumour and normal tissues. Each point represents an individual patient. The solid line represents a linear regression fitted for descriptive purposes only. Spearman’s correlation coefficient (r_s_) and corresponding *p*-value are indicated.

To determine whether altered promoter methylation was associated with changes in *BRCA1* expression, *BRCA1* messenger ribonucleic acid (mRNA) levels were quantified in tumour and matched normal tissues using quantitative reverse transcription PCR (qRT-PCR). Although *BRCA1* expression tended to be reduced in tumour tissues compared with normal counterparts, this difference did not reach statistical significance (Figure 4A). Notably, correlation analysis revealed a significant inverse association between tumour-specific *BRCA1* promoter methylation changes and *BRCA1* mRNA expression (Figure 4B). This suggests that, while *BRCA1* expression is not uniformly altered across the cohort, promoter hypermethylation may contribute to epigenetic repression of *BRCA1* in a subset of tumours.

## 4. Discussion

In the current study, we investigated the methylation status of the *BRCA1* promoter in breast tumours from a cohort of Black South African women and examined its relationship with gene expression and clinicopathological characteristics. We demonstrate that breast tumours exhibit increased *BRCA1* promoter methylation across five interrogated CpG sites compared with matched normal tissue. Importantly, these tumour-associated methylation changes were enriched in hormone receptor-negative disease and TNBC and were inversely correlated with *BRCA1* mRNA expression in a subset of tumours. Together, these findings suggest the presence of tumour-associated epigenetic changes that may influence *BRCA1* regulation in this population.

Breast cancer is a biologically heterogeneous disease, with substantial variation in tumour behaviour, molecular drivers, and treatment outcomes across populations. While studies conducted in high-income countries have yielded major advances in biomarker discovery and targeted therapies, comparable molecular data from African populations remain limited. Accumulating evidence indicates that breast cancer in women of African ancestry is characterised by a higher prevalence of aggressive subtypes, including TNBC, younger age at diagnosis, and poorer survival outcomes, likely reflecting a complex interplay between genetic ancestry, environmental exposures, and contextual factors [40,41,42]. Despite the implementation of standardised treatment protocols by the South African Department of Health [43], mortality rates among Black South African women remain disproportionately high [44], underscoring the urgent need for population-specific molecular studies to inform risk stratification and therapeutic decision-making.

*BRCA1* and *BRCA2* encode proteins that play central roles in the DNA damage response, particularly in the repair of DNA double-strand breaks via homologous recombination. Loss of expression or function of these proteins has long been associated with aggressive breast cancer phenotypes, with the most extensively characterised underlying mechanisms being the presence of pathogenic germline or somatic sequence variants resulting in impaired protein function or reduced transcript stability [10,45]. In a clinical setting, *BRCA* mutations are used as a marker to guide the therapeutic use of DNA-damaging agents, including platinum-based chemotherapy and poly(ADP-ribose) polymerase (PARP) inhibitors, as they are predictive of high response rates and prolonged progression-free survival in neoadjuvant and metastatic settings, respectively [46,47]. More recently, promoter hypermethylation, most notably of *BRCA1*, has emerged as an alternative mechanism of gene silencing in sporadic breast cancer, giving rise to “BRCA-like” tumours that phenocopy the homologous recombination deficiency observed in *BRCA1*-mutated cancers [22,23,48,49]. On this basis, the potential applicability of platinum-based or PARP inhibitor therapy for tumours harbouring *BRCA1* promoter methylation has become an area of active investigation, with studies reporting conflicting findings. Experimental studies using cell line and patient-derived xenograft models of *BRCA1*-methylated breast and ovarian cancers have demonstrated sensitivity to platinum-based chemotherapy and PARP inhibitors, with similar associations reported in some population-based studies [24,25,26,27,28,29,30,37,50]. In contrast, other studies, including larger meta-analyses, have reported that *BRCA1* methylation does not reliably predict platinum sensitivity and may even contribute to acquired resistance, possibly reflecting the reversible nature of epigenetic *BRCA1* silencing during treatment [31,32,33,34]. Therefore, although *BRCA1* promoter methylation has been proposed by some as a predictive biomarker for DNA-damaging therapies, its clinical utility may be limited compared with the more robust predictive value of germline *BRCA1*/2 mutations.

Consistent with the known link between *BRCA1* dysfunction and basal-like, triple-negative breast cancer, we observed higher *BRCA1* promoter methylation in TNBC compared with Luminal A tumours, and in ER- and PR-negative tumours compared with hormone receptor-positive disease. These findings collectively support a role for epigenetic *BRCA1* silencing in shaping hormone receptor-negative tumour phenotypes [51,52,53,54,55,56]. A plausible mechanistic explanation for the ER-negative phenotype observed in *BRCA1*-deficient tumours is the positive regulation of *ERα* expression by *BRCA1*. Indeed, *BRCA1* is recruited to the *ERα* promoter via the transcription factor OCT1, where it activates *ERα* transcription [57]. Given that *PR* expression is a downstream target of functional ER signalling in breast cancer, reduced *ER* transcriptional activity may secondarily lead to loss of *PR* expression [58]. In addition to this ER-dependent mechanism, *BRCA1* has also been shown to regulate PR activity directly. Specifically, Calvo and Beato (2011) demonstrated that *BRCA1*, in complex with *BARD1*, functions as an E3 ubiquitin ligase that promotes ligand-independent and ligand-dependent PR ubiquitination and degradation, thereby limiting PR abundance and recruitment to target gene promoters [59]. Following hormone stimulation, the *BRCA1*/*BARD1* complex is also recruited to progesterone-responsive elements, where it modulates local chromatin structure through histone H2A monoubiquitination, contributing to epigenetic silencing of PR target genes [59]. Together, these mechanisms provide a biological framework through which *BRCA1* silencing may lead to concurrent ER and PR negativity observed in tumours from our population. Intriguingly, our findings also revealed that *BRCA1* methylation was not associated with HER2 status, supporting previous reports that *BRCA1* methylation-driven tumourigenesis preferentially aligns with hormone receptor-negative pathways rather than HER2 amplification [60].

Only one patient in this cohort harboured a documented *BRCA1* mutation. This observation is consistent with previous reports suggesting that *BRCA1* promoter methylation and *BRCA1* mutations rarely co-occur [28]. While exploratory, these findings may offer insight into BRCA-like breast cancers observed in Black South African women, where pathogenic BRCA variants are infrequently detected despite tumours exhibiting clinical and pathological features similar to hereditary breast cancers [40,42,61].

This study has several strengths. To the best of our knowledge, it is the first to examine *BRCA1* methylation and gene expression in an exclusively South African cohort of women with breast cancer. By analysing matched tumour and adjacent normal tissues from the same individuals, the study controlled for potential confounding biological and lifestyle factors, such as age and diet. Several limitations should be acknowledged. Both tumour and matched normal breast tissues are a heterogeneous mixture of cells, containing epithelial, stromal, and immune cells in differing proportions, which may influence the *BRCA1* methylation profiles reported in this study. While approaches exist to adjust for cellular heterogeneity, these were not feasible due to limited tissue availability and the lack of validated reference datasets for breast tissue in South African populations [62]. Moreover, although control samples were excised approximately 5 cm from the primary tumour margin and were independently reviewed by a qualified pathologist to confirm the absence of invasive carcinoma or in situ disease, we cannot exclude the possibility of molecular alterations reflective of pre-malignant change. Indeed, recent studies have shown that histologically normal breast tissue adjacent to tumours harbour DNA methylation changes extending beyond the visible tumour boundary [63]. Such effects may reflect early clonal alterations in precurser cells that are not detectable by routine histopathology. If present, these changes could skew tumour-normal differences in *BRCA1* methylation, potentially leading to over- or underestimation of the true magnitude of tumour-specific alterations in *BRCA1* methylation. Future studies incorporating reduction mammoplasty tissue or biopsies from the adjacent healthy breast may help further delineate tumour-specific versus field-associated methylation changes. The cross-sectional design of our study also precludes conclusions about causality, and whether *BRCA1* promoter methylation contributes to tumour development or reflects a secondary change requires investigation in longitudinal studies. Although pyrosequencing is a highly sensitive and accurate method and considered the “gold standard” for quantifying DNA methylation, it is limited to assessing relatively small genomic regions within a single assay. Consequently, only a small segment of the *BRCA1* promoter could be interrogated in the present study, encompassing six CpG sites located approximately 861–901 bp upstream of the transcription start site. These CpG sites either span or flank putative SP1 and CREB-binding protein (CREBP1) transcription factor binding sites, which may support a regulatory role for methylation at these CpGs. Consistent with this possibility, methylation at the interrogated CpG sites showed significant correlations with *BRCA1* gene expression, suggesting functional relevance. However, we acknowledge that our analysis may not capture methylation changes in other regulatory regions of *BRCA1*, such as intronic regulatory elements or additional CpG islands that may influence gene expression. Finally, given our modest sample size, our study was underpowered to adjust for potential confounders including obesity, HIV infection, and metabolic disease. Although obesity has been reported to influence DNA methylation, we observed no significant correlation between *BRCA1* methylation and BMI. Similarly, stratification by comorbidity revealed no significant association between tumour-normal differences in *BRCA1* methylation and HIV or metabolic disease (Table S1).

## 5. Conclusions

Our study provides the first comprehensive analysis of *BRCA1* promoter methylation and gene expression in breast tumours from South African women. We demonstrate that *BRCA1* promoter hypermethylation is enriched in hormone receptor-negative and triple-negative breast cancers and is inversely correlated with *BRCA1* expression in a subset of tumours, supporting a role for epigenetic silencing in driving aggressive tumour phenotypes in this population. These findings further highlight that *BRCA1* promoter methylation occurs largely independently of pathogenic gene mutations, addressing a key question regarding “BRCA-like” cancers in women who test negative for *BRCA* variants. Collectively, our results underscore the potential of *BRCA1* methylation as a clinically relevant biomarker for identifying patients who may benefit from targeted therapies. Future studies in larger, longitudinal, and ethnically diverse cohorts are warranted to validate these observations and to further clarify the mechanistic and therapeutic implications of *BRCA1* epigenetic dysregulation in African populations.

## Figures and Tables

**Table 1:** Demographic, histomorphological, and clinical characteristics of study participants.

Clinical Characteristic	Mean (sd)	%	Mean (sd)	%	Mean (sd)	%
Age at diagnosis	Early (≤49)		Late (≥50)			
	41.5 (±6.9)	59.2	61.4 (±8.4)	40.7		
BMI	Normal weight		Overweight		Obese	
	22.9 (±1)	18.5	27.6 (±1)	22.2	37.3 (±7)	59.2
Menarche *	Early (<12)		Normal (12–14)		Late (>14)	
	11	3.7	13 (±0.8)	48.1	16.1 (±1)	40.7
Parity	Nulliparous		Low parity (≤5)		High parity (≥5)	
	0	14.8	2.2 (±1)	77.8	7 (±1)	7.4
Maternal age *	<20		20–29		≥30	
	16.5 (±1.8)	29.6	24.9 (±2.4)	40.7	32 (±2)	7.4
Menopause	Premenopausal		Postmenopausal			
	-	55.6		44.4		

* Indicates missing data.

**Table 2:** Clinical and histomorphological patient characteristics.

Clinical Characteristic		*n*	%
Disease type	Invasive ductal carcinoma	22	66.7
	Infiltrating ductal carcinoma	1	14.8
	Papillary breast carcinoma	1	3.7
	Mucinous breast cancer	3	11.1
Disease stage	II	13	48.1
	III	14	51.9
Molecular subtype	Luminal A	7	25.9
	Luminal B	16	59.2
	Triple-negative/Basal-like	4	14.8
Tumour grade	I	9	29.6
	II	14	51.9
	III	4	18.5
Chemotherapy	EC	1	3.7
	EC + Taxane	11	40.7
	EC + Taxane + Herceptin	5	18.5
Treatment response	Near total effect	4	23.5
	Partial response	5	29.4
	Stable disease	7	41.2
*BRCA1*/2 mutations	*BRCA1* Positive	1	3.7
	*BRCA2* Positive	2	7.4
	Negative	21	77.8

## Data Availability

Raw DNA methylation and *BRCA1* gene expression data generated in this study are provided in Supplementary Table S2.

## Supplementary Materials

The following supporting information can be downloaded at https://www.mdpi.com/article/10.3390/genes17040407/s1. Table S1: Association between clinical comorbidities and tumour–normal differences in BRCA1 methylation. Table S2: CpG-specific and cumulative BRCA1 promoter methylation levels and relative BRCA1 gene expression in paired breast tumour and adjacent normal tissues from South African patients.

## Abbreviations

BC	breast cancer
BARD1	*BRCA1*-associated RING domain protein 1
BMI	body mass index
*BRCA1*	breast cancer susceptibility gene 1
*BRCA2*	breast cancer susceptibility gene 2
BREC	Biomedical Research Ethics Committee
cDNA	complementary DNA
CpG	cytosine–phosphate–guanine
CREBP1	CREB-binding protein
DCIS	ductal carcinoma in situ
DNA	deoxyribonucleic acid
dx	diagnosis
EC	epirubicin and cyclophosphamide
ER	oestrogen receptor
ERα	oestrogen receptor alpha
HER2	human epidermal growth factor receptor 2
HIV	human immunodeficiency virus
HS	high sensitivity
IALCH	Inkosi Albert Luthuli Central Hospital
Ki67	Ki-67 proliferation index
mRNA	messenger ribonucleic acid
NACT	neoadjuvant chemotherapy
OCT1	octamer transcription factor 1
PARP	poly(ADP-ribose) polymerase
PCR	polymerase chain reaction
PR	progesterone receptor
qRT-PCR	quantitative reverse transcription polymerase chain reaction
RIN	RNA integrity number
RNA	ribonucleic acid
SD	standard deviation
SP1	Specificity Protein 1
TNBC	triple-negative breast cancer
