# Supplementary material for: BRCA1 Promoter CpG Methylation in Breast Cancer: A Pilot Study in African Women
Source: Genes (Basel). 2026 Mar 31;17(4):407. doi: 10.3390/genes17040407 (PMC13117029; doi:10.3390/genes17040407)
Supplement: Supplementary file 1 [file genes-17-00407-s001.zip › genes-4163908-supplementary.pdf]

Table S1. Association between clinical comorbidities and tumour–normal differences in BRCA1 methylation.

Variable	Category	n	Δ Methylation (%)	p-value
**Metabolic disease**	Yes	10	3.75 (−4.17 – 18.67)	0.56
	No	17	13.17 (2.50 – 21.50)	
**HIV status**	Positive	13	13.83 (2.33 – 21.67)	0.85
	Negative	14	7.00 (1.50 – 19.04)	

Data presented as median and interquartile range (25–75th percentile) of Δ methylation between tumour and paired adjacent tissues

Table S2. CpG-specific and cumulative BRCA1 promoter methylation levels and relative BRCA1 gene expression in paired breast tumour and adjacent normal tissues from South African patients.

	BRCA1 Methylation (%)														Relative *BRCA1* expression	
Patient	CpG 1		CpG2		CpG3		CpG4		CpG5		CpG6		Cumulative			
	N	T	N	T	N	T	N	T	N	T	N	T	N	T	N	T
1	1	20	0	35	31	44	32	38	0	43	30	44	16	37	8.037	1.715
2	5	29	6	51	33	78	5	51	0	58	86	67	23	56	0.768	0.000
3	38	47	50	48	63	69	55	65	60	32	52	63	53	54	0.575	1.141
4	20	10	39	45	55	57	44	52	50	55	61	77	45	49	0.560	0.572
5	26	38	66	59	79	75	71	69	74	76	63	80	63	66	0.000	1.922
6	23	40	41	65	60	64	51	67	60	95	67	91	50	70	0.785	0.975
7	34	40	59	89	68	93	62	91	69	92	74	90	61	83	†	†
8	31	31	47	46	57	58	49	46	58	57	66	64	51	50	0.576	0.662
9	26	23	50	64	64	77	57	67	61	72	64	59	54	60	†	†
10	13	15	35	29	47	43	43	33	49	44	59	44	41	35	0.000	0.719
11	25	55	0	73	40	86	17	77	17	86	38	77	23	76	1.360	0.671
12	21	22	40	48	57	66	17	64	56	67	62	75	42	57	1.132	1.058
13	49	44	68	49	87	71	81	56	87	62	73	33	74	53	0.000	1.688
14	25	28	65	43	78	59	67	56	70	57	59	62	61	51	1.083	1.615
15	25	22	65	42	79	58	71	52	71	57	57	65	61	49	0.963	0.905
16	44	20	34	45	43	62	32	51	55	61	13	65	37	51	†	†
17	26	16	47	52	60	65	48	45	57	64	65	76	51	53	0.975	0.933
18	1	22	21	45	23	59	0	51	22	58	22	67	15	50	0.793	0.532
19	25	1	50	71	68	72	54	100	63	96	76	93	56	72	0.000	0.774
20	12	19	30	33	41	44	32	33	38	42	49	45	34	36	0.592	1.019
21	18	31	35	51	49	65	43	57	47	65	60	71	42	57	0.742	0.354

22	18	19	36	38	44	49	36	41	41	55	44	61	37	44	†	†
23	24	34	41	52	56	63	54	55	56	63	63	67	49	56	6.630	0.000
24	37	21	56	38	74	50	67	48	69	50	74	58	63	44	0.792	0.000
25	11	33	23	55	32	74	28	51	31	71	36	75	27	60	0.405	0.630
26	31	1	56	65	71	97	67	81	65	97	65	93	18	52	1.923	0.000
27	11	24	16	46	20	59	18	54	23	58	22	71	59	72	0.637	0.509

Data are expressed as percentage (%) methylation at each investigated BRCA1 promoter CpG site determined by pyrosequencing. Cumulative methylation (%) represents the mean across the six analysed CpG sites per sample. Relative BRCA1 expression was measured by qRT-PCR and normalised to the reference genes, RPLP0 and SYMPK. † Gene expression data not available for these samples.

Abbreviations: N, histologically normal adjacent breast tissue; T, matched breast tumour tissue; BRCA1, Breast Cancer 1; CpG, cytosine–phosphate–guanine dinucleotide.
